# Opioid Receptor-Mediated and Non-Opioid Receptor-Mediated Roles of Opioids in Tumour Growth and Metastasis

**DOI:** 10.3389/fonc.2021.792290

**Published:** 2021-12-23

**Authors:** Claudia A. Scroope, Zane Singleton, Markus W. Hollmann, Marie-Odile Parat

**Affiliations:** ^1^ School of Pharmacy, The University of Queensland, St Lucia, QLD, Australia; ^2^ Department of Anaesthesiology, Amsterdam University Medical Center, Academic Medical Center (AMC), Amsterdam, Netherlands

**Keywords:** opioid receptor, cancer, metastasis, TLR4, OGFr, opioid antagonist

## Abstract

Opioids are administered to cancer patients in the period surrounding tumour excision, and in the management of cancer-associated pain. The effects of opioids on tumour growth and metastasis, and their consequences on disease outcome, continue to be the object of polarised, discrepant literature. It is becoming clear that opioids contribute a range of direct and indirect effects to the biology of solid tumours, to the anticancer immune response, inflammation, angiogenesis and importantly, to the tumour-promoting effects of pain. A common misconception in the literature is that the effect of opioid agonists equates the effect of the mu-opioid receptor, the major target of the analgesic effect of this class of drugs. We review the evidence on opioid receptor expression in cancer, opioid receptor polymorphisms and cancer outcome, the effect of opioid antagonists, especially the peripheral antagonist methylnaltrexone, and lastly, the evidence available of a role for opioids through non-opioid receptor mediated actions.

## Introduction

Opioids are administered to cancer patients to manage the pain associated with the disease, its treatment and in palliative care. The possibility that opioids may alter the course of cancer is therefore of high clinical relevance. Opioids given to cancer surgery patients in the perioperative period are of particular interest because despite the short time frame when they are administered, they have been hypothesised to contribute, together with a number of other optimisable variables, to long-term cancer outcome (1, 2).

*In vitro* and animal studies evaluating the influence of opioids on tumour growth and metastasis are abundant and highly discrepant, as reviewed elsewhere (3–5). A systematic review and meta-analysis of the ability of analgesic drugs to reduce metastasis in experimental cancer models concluded that opioids did not show a significant effect on the incidence of metastasis (6). Clinical studies have compared recurrence after cancer surgery employing regional anaesthesia and analgesia techniques, which allow pain control while reducing opioid exposure. Available at this time are a meta-analysis of several retrospective studies (7), randomised studies analysed *a posteriori* for cancer outcomes (8, 9), and a large-scale prospective randomised clinical trial comparing the suggested most tumour-protective anaesthetic strategy (regional anaesthesia and total intravenous anaesthesia) versus the proposed most tumour-promoting strategy (opioids plus volatile anaesthetics) (10). These studies, which are not designed to test the effect of opioids per se, use opioid-sparing techniques that have independent tumour modulating actions, and tend to combine disparate cancer types and patient-specific tumour genomics, have not elucidated whether opioids modulate the cancer-specific outcomes of surgery.

Numerous factors are likely to contribute to the lack of a net effect of opioids on tumour growth and metastasis *in vivo*. Opioids directly affect cancer cells as well as stromal cells that are key to the control of tumour growth and metastasis, especially immune and endothelial cells, and their pro-invasive paracrine cell-cell interaction (11, 12). The actions of opioids can result from their central or peripheral activity, at opioid as well as non-opioid receptors such as Toll-like receptor 4, which is abundantly expressed on immune cells and some cancer cells (13). In addition, the effect of opioids may indirectly stem from modulation of neuroendocrine responses, inflammation, stress and pain, all of which modulate tumour development (2). Opioids are broadly immunosuppressive and lower cellular and humoral responses, which may be of clinical relevance (1). However, because pain itself is immunosuppressive and promotes tumour growth and metastasis, opioids are protective against tumour growth and metastasis in animal models that incorporate pain (14, 15). Another variable factor is that not all opioids are equal, and their pharmacokinetic and pharmacodynamic characteristics may lead to differences in tumour-modulating properties. There is data to suggest that administration of opioids leading to continuous *versus* discontinuous opioid receptor activation may have different effects on cancer-relevant parameters (16, 17).

In view of these complexities, the present review seeks to distinguish the role of opioid analgesics from the role of opioid receptors particularly the mu-opioid receptor, the major target of the analgesic effect of this class of drugs. We review the evidence on increased opioid receptor expression in tumours, the literature investigating opioid receptor polymorphisms and cancer outcome, and the effect of opioid antagonists, especially the peripheral antagonist methyl naltrexone (MNTX). Finally, we suggest that the effects of opioids on non-opioid receptors, including but not limited to TLR4, may offer novel insights into the role of opioids on cancer in the future.

## Opioid Receptor Expression in Various Cancer Types

We searched the literature for studies comparing the expression of opioid receptors in cancer cells or tissues to that of relevant, healthy control cell or tissues. We used the following search protocol in PubMed: (“opioid receptor expression”) AND (((cancer) OR (tumour)) OR (tumor)) and screened all the hits produced by the search up to September 10^th^, 2021, further adding relevant studies that were cited by these articles. The results are compiled in **Table 1**.

**Table 1:** Studies documenting altered opioid receptor expression in cancer.

Receptor	Cancer	Sample	Mode of Detection	Findings	Result	Reference
μ-opioid receptor	Metastatic Lung Cancer	Tissue samples	Immunohistochemistry	μOR expression was increased significantly in cancer samples from patients with lung cancer compared with adjacent control tissue	μOR ↑	Singleton (18)
μ-opioid receptor	Laryngeal Carcinoma	Tissue samples	Immunohistochemistry	μOR staining intensity was significantly increased in laryngeal-carcinoma compared to the adjacent normal tissue	μOR ↑	Lahav (19)
μ-opioid receptor	Laryngeal squamous cell carcinoma (LSCC)	Tissue samples	Immunohistochemistry	μOR levels in tumour tissues were significantly higher than those in adjacent non-tumour tissue There was a statistically significant relationship between high μOR and low disease-free survival	μOR ↑	Zhang (20)
μ-opioid receptor	Lung Cancer	Murine Lewis Lung Carcinoma (LLC) cells, various human non-small cell lung cancer (NSCLC) cells and non-tumorigenic human BEAS-2B cells	Immunohistochemistry	μOR expression levels were higher in bronchioloalveolar carcinoma, adenocarcinoma and, to a lesser extent, squamous cell carcinoma than in normal lung samples	μOR ↑	Matthew (21)
μ-opioid receptor	Prostate Cancer	Tumour specimens from 113 patients with Stage IV prostate cancer and samples of benign prostatic hyperplasia (BPH) used as controls	Laser scanning confocal microscopy. Images were processed to quantitate μOR-ir as a marker of μOR expression	Tumour samples had a greater μOR expression than BPH control samples. In samples with high μOR expression, the receptor was generally localised to the membrane, whereas in samples with low expression it was internalised (perhaps inactive)	μOR ↑	Zylla (22)
μ-opioid receptor	Stage I-III Pancreatic Ductal Adenocarcinoma	Tissue Samples	Immunohistochemistry	There was no significant differences between μOR expression levels in the tumour and adjacent non-tumour tissues	–	Zhang (23)
μ and κ-opioid receptors	Breast cancer	Tissue samples	Immunohistochemistry	No difference in μOR or κOR between primary tumour, peritumoral area and lymph node metastasis Significantly less κOR in lymph node samples from patient with recurrence vs patients with no recurrence	–	De Sousa (24)
δ–opioid receptor	Lung Cancer	SCLC cell lines (SCLC-22H and 16HC) and NSCLC cell line (NCI-23)	RT-PCR binding assay and DNA sequence analysis	mRNA expression of δ–OR detected in all five SCLC but low level in NSCLC cell line and none in in normal lung tissue or cultured lung fibroblasts DNA seq. analysis of SCLC-22H, 16HC and NCI-23 revealed that the δOR was not mutated	δOR ↑	Schreiber (25)
δ–opioid receptor	Breast cancer	Cultured cells, (cancer: MCF-7, MDA-MB-231, SKBR-3 and epithelial MCF-10F) Patient tissue sections	RT-PCR Western blot	δOR mRNA and protein expression was significantly higher in breast cancer tissues than in the corresponding paracancerous tissues δOR expression was positively correlated with tumour metastasis, clinical stage, and poor prognosis	δOR ↑	Wei (26)
δ–opioid receptor	Hepatocellular Carcinoma	Cultured cells (LO2, HepG2, and Hep3B) and tissue sections	RT-PCR Western blot Immunohistochemistry	δOR mRNA and protein levels higher in HCC lesions than in the adjacent tissues and normal liver tissues Results also indicate that activation of δOR promotes HCC cell proliferation	δOR ↑	Tang (27)
δ-opioid receptors	Lung cancer	SCLC and NSCLC cell lines, normal mouse lung membranes	Radiolabelled δ-opioid receptor ligand binding	6 SCLC but not NSCLC cell lines or normal mouse lung membranes showed δ-opioid receptor binding (specifically of the δ2 subtype)	δOR ↑	Campa (28)
μ and δ-opioid receptors	Lung Cancer	Human cancer patients, *in vivo* imaging	PET analysis of kinetics and distribution of binding of δOR- and μOR- binding tracers C-MeNTI and C-CFN	μOR and δOR were significantly μOR e abundant in lung carcinoma than in the normal host tissue Relative increase in δOR in malignant lesions is greater than that of the μOR	μOR ↑ δOR ↑	Madar (29)
μ, δ, and κ-opioid receptors	Stage I-III Triple Negative Breast Cancer	Publicly available bulk RNA-seq data	RNA-seq analysis of tissue or single cells	μOR expression extremely low in both cancer and normal tissue κOR, δOR and OGFR expression higher in tumour vs normal tissue κOR mostly on cancer cells, OGFR mostly on immune cells	δOR ↑ κOR ↑	Montagna (30)
μ, δ, and κ-opioid receptors	Oesophageal Squamous Cell Carcinoma (ESCC)	Cultured cells and tissue sections	Flow cytometry Immunocytochemistry Western blot Immunohistochemistry	All OR receptors expressed in ESCC cell lines, to varying degree κOR membrane, cytoplasmic and nuclear localisation detected κOR expression increases with tumour grade. Nuclear κOR correlates with lymph node metastasis and poor prognosis	κOR ↑	Zhang (31)
Opioid Growth Factor receptor (OGFr)	Pancreatic and colon cancer	Human PaCa-2, BxPC-3, Capan-2 (pancreatic) HT-29, HCT 116 (colon) inoculated into nude mice	Receptor binding analysis Northern blot	No differences in receptor binding or gene expression between cancer cells *in vitro* and small, medium, or large tumours	–	Zagon (32)

Altered opioid receptor expression in cancer has been observed as early as 1996 (28). Changes in expression of μ, δ, or κ-opioid receptors in cancer cells or tissues are reported in various cancer types and overall, point to an increased expression in cancer. The majority of studies described an increase in μOR (18–22), while some reports showed no significant differences in μOR expression levels between the tumour and adjacent non-tumour tissues (23, 24). No studies reported a decreased expression of OR in cancer.

While μOR has long been the focus of attention in the tumour microenvironment, δOR also showed an increase in expression compared to normal tissue (25–30), and interestingly, it is reported that the relative increase in δOR in malignant lesions is greater than that of the μOR (29, 30). While κOR has not been investigated as extensively as other receptors in the family, an increase in expression from healthy to cancerous tissue has been reported (30, 31).

Importantly, some studies attempted to link the level of OR expression in tumours to cancer aggressiveness or prognosis. For example, increased μOR in tumour tissue was associated with worse progression-free and overall survival in patients with metastatic, hormone sensitive prostate cancer (22) and low disease-free survival in laryngeal squamous cell carcinoma (20). Additionally, δOR expression was positively correlated with tumour metastasis, and receptor activation promoted proliferation in breast cancer cells (26). Similarly, κOR expression increases with tumour grade in oesophageal squamous cell carcinoma (31). In addition to the three commonly explored opioid receptors, McLauglin et al. investigated the expression of opioid growth factor receptor (OGFr), revealing no differences in receptor binding or gene expression between cancer cells *in vitro* and in small, medium, or large tumours (33).

Overall, the literature indicates increased expression of OR in cancer compared to healthy tissue, and links increased expression to stronger cancer aggressiveness. To evaluate whether OR expression may have a causal relationship with aggressiveness, we next reviewed studies where manipulation of OR expression was carried out and aggressive features measured as a readout.

## Effect of Opioid Receptor Manipulation on Tumour Growth and Aggressiveness

A number of studies investigated the effect of experimentally manipulating opioid receptor expression, e.g. through overexpression, mRNA silencing or gene disruption, in an attempt to demonstrate causality between receptor expression and change in tumour aggressive features. These studies are summarised in **Table 2**. It is important to note that few studies provide insight in the role of receptor expression *in vivo*, especially in the clinical setting.

**Table 2:** Effect of experimental manipulation of opioid receptor expression on tumour growth and aggressiveness.

Receptor	Cancer	Manipulation/comparison	Findings	Reference
μ-opioid receptor	Squamous Cell Carcinoma of the Head and Neck	siRNA down regulation of μOR in FaDu MDA686Tu and UMSCC47 cultured cells	In FaDu and MDA686Tu cells, downregulating μOR expression inhibited aggressive features	Gorur A (34)
μ-opioid receptor	Lung Cancer	Lewis lung carcinoma (LLC) cells were either transfected with control shRNA or μOR -1 shRNA	Silencing (shRNA) μOR expression in LLC inhibits invasion and anchorage-independent growth *in vitro*, and experimental metastasis *in vivo*. μOR -deficient mice inoculated with LLC have reduced tumour formation.	Matthew (21)
μ-opioid receptor	Melanoma	Wild-type and μOR R-deficient mice were inoculated with B16 melanoma cells that secrete endogenous mu-opioid peptides	μOR -deficient mice demonstrated a marked reduction in tumour growth and significantly higher infiltration of immune cells into the tumours when inoculated with B16 melanoma cells	Boehncke (35)
μ-opioid receptor	Lung Cancer	μOR -overexpressing lung cancer xenografts in nude mice μOR was inhibited with the peripheral μOR antagonist MNTX	Overexpression of μOR in cancer cells increased primary tumour growth rates and lung metastases Inhibiting μOR attenuates EGF-induced proliferation and migration	Lennon (36)
μ-opioid receptor	Non-Small Cell Lung Cancer	Stable vector control and μOR 1 overexpressing human bronchioloalveolar carcinoma cells Xenografted to tumour-bearing nude mice	μOR overexpression increased proliferation and extravasation *In vivo*, overexpression of μOR in human bronchoalveolar carcinoma cells increased primary tumour growth rates in nude mice by approximately 2.5-fold and lung metastasis by approximately 20-fold compared with vector control cells	Lennon (37)
δ-opioid receptor	Breast Cancer	MCF7 cells transduced with δOR siRNA or control siRNA Xenografted to tumour-bearing nude mice	δOR siRNA inhibits tumour growth *in vitro* and *in vivo*	Wei Y-C (26)
δ-opioid receptor	Hepatocellular Carcinoma	siRNA down regulation of δOR in cultured cells (LO2, HepG2, and Hep3B)	δOR siRNA inhibits aggressiveness *in vitro* and *in vivo*	Tang (27)
μ and δ-opioid receptors	Non-small Cell Lung Cancer	H2009 Non-small Cell Lung Cancer cell line	siRNA down regulation of either μOR and δOR decreases EGFR activation by EGF and EGFR transactivation by morphine	Fujioka (38)
Opioid Growth Factor receptor (OGFr)	Squamous Cell Carcinoma of the Head and Neck	human SCC-1 and clonal lines overexpressing wild type OGFR or empty vector	OGFR overexpression led to decreased cell proliferation	McLaughlin P (33)

The literature suggests a cancer-promoting role for the μOR in both cancer and non-cancer cells that support tumour growth. The siRNA downregulation of μOR inhibited aggressive features in lung cancer and squamous cell carcinoma of the head and neck *in vitro* (21, 34, 36). Downregulation of μOR also decreased tumour take in the lungs after IV inoculation (21). Conversely, μOR overexpression promoted lung cancer cell line aggressiveness *in vitro* (36), and *in vivo* analysis of μOR-overexpressing human bronchioloalveolar carcinoma cells xenografted to nude mice showed a marked increase in tumour growth rates compared to the stable vector-transfected control group (37). Interestingly, overexpressing the μOR led to increased *in vitro* (36) and *in vivo* (37) tumour growth or aggressiveness in the absence of any opioid added to culture medium or administered to mice. Furthermore, μOR gene-disrupted mice had markedly reduced tumour formation compared to their wild type counterparts when inoculated with μOR expressing lung cancer cells, implying a role for the μOR in host cells (21). This was further demonstrated in experiments where μOR-deficient mice or wild type controls were inoculated with B16 melanoma cells (35). The μOR-deficient mice demonstrated a marked reduction in tumour growth compared to wild-type and the authors suggested this was due to inhibition by melanoma-secreted β-endorphin of infiltration and proliferation of anti-cancer immune cells (35).

Two independent studies on δOR siRNA showed that silencing the receptor inhibited tumour growth *in vitro* and *in vivo* in both breast (26) and liver (27) cancer. Another study on both μOR and δOR expression in lung cancer revealed that siRNA down regulation of either receptor decreased EGFR activation by EGF and EGFR transactivation by morphine (38).

Combined with the findings from **Table 1**, this would suggest that not only μOR and/or δOR expression is increased in cancerous cells or tissue, but overexpression also increases tumour growth and metastases. It is important to note that if ORs modulate tumour biology *via* activation by opioid, then their level of expression should be studied together with opioid dose-response, both *in vitro* and *in vivo*. If OR overexpression has functional consequences promoting tumour growth or metastasis, then OR mutations or polymorphisms that affect receptor function should lead to altered cancer outcomes.

## Opioid Receptor Polymorphisms and Cancer Outcome

We next reviewed the existing literature assessing opioid receptor polymorphisms (**Table 3**). The A118G single-nucleotide polymorphism (SNP) of the μOR is the most frequent of all the μOR (OPRM1) gene variants, resulting in an amino acid change from asparagine to aspartate at position 40 of the μOR, which leads to altered signalling and expression of the receptor (45, 46). The literature indicates that having the G allele results in higher (42), or lower (43, 44) cancer risk, depending on the studies (different population; breast cancer or oesophageal cancer), while having the G allele resulted in better (41) or unchanged (39, 40) outcomes after breast cancer surgery. These studies therefore do not provide a clear association between μOR function and cancer, however, it must be kept in mind that a number of confounders might be at play, since patients carrying the G allele require higher opioid doses for pain management (46) and this polymorphism may also modulate drinking and smoking behaviours (45). We may see more studies evaluating genetic alteration of μ or other OR function and cancer in the future, but at present the evidence of a link between altered function of μOR and cancer outcome is inconsistent.

**Table 3:** Opioid receptor polymorphisms and cancer outcome.

Receptor and mutation	Cancer	Population	Method	Findings	Reference
μ-opioid receptor gene A118G SNP (AA, AG, GG genotype)	Breast cancer	Breast cancer or benign biopsies from Korean women n= 200	Genotype determined *via* PCR of blood sample	Breast tumour recurrence was not influenced by A118G genotype in Korean women	Lee (39) Oh (40)
6 μ-opioid receptor gene polymorphisms including A118G (AA, AG, GG genotype)	Breast cancer	Breast cancer biopsies from 766 African American and 1,273 European American women n= 2039	Genotype determined *via* PCR of blood sample	Of the six polymorphisms studied, the only one with a statistically significant impact on mortality was A118G Ten-year mortality was reduced in patients with at least one variant G allele at A118GBeing heterozygous for AG genotype was significantly protective over being homozygous for AA, with a reduced mortality rate of 9% compared to 18% GG genotype at A118G was uncommon in the studied population, precluding significant conclusions	Bortsov (41)
μ-opioid receptor A118G polymorphism	Breast Cancer	North-eastern Polish females recently diagnosed with breast cancer n = 741	Genotype determined *via* PCR of blood sample	G allele presence is strongly associated with increased breast cancer incidence	Cieślińska (42)
μ-opioid receptor A118G polymorphism	Oesophageal Squamous Cell Carcinoma	Chinese population 490 ESCC patients and 470 control subjects n= 960	Genotype determined *via* PCR of blood sample	The frequency for the A allele of A118G was significantly higher in ESCC cases There was also a significant interaction between the A allele of A118G and current smoking or alcohol consumption Current smokers or drinkers with the A allele have the highest OSCC risk	Xu (43)
μ-opioid receptor A118G polymorphism	Oesophageal Squamous Cell Carcinoma	Male and female OSCC patients from Chinese population n = 551	Genotype determined *via* PCR of blood sample	AA genotype was associated with a significantly higher rate of OSCC	Wang (44)

## Effect of Opioid Antagonists on Tumour Growth and Metastasis: Preclinical Studies and Clinical Approaches

The role of opioid agonists *in vitro* and *in vivo* in preclinical studies has been evaluated and several reviews have concluded that opioids neither favour nor prevent cancer (5, 47, 48, 49–51) and elaborated on the potential reasons for the discrepancy (4). Due to this previous coverage, in the present review the effect of opioid agonists is deliberately not covered, however the next section will review the literature on opioid antagonists and cancer.

An *in vitro* study established that MNTX inhibits VEGF-induced activation of VEGF receptors 1 and 2 and consequent endothelial cell migration and proliferation, *in vitro* hallmarks of angiogenesis (52). MNTX similarly attenuated EGF-induced proliferation and migration of cultured human H358 NSCLC cells in a dose-dependent manner (36). MNTX exhibited synergistic effects when applied to cultured endothelial cells in combination with the antimetabolite 5-Fluorouracil and the VEGF-monoclonal antibody Bevacizumab. The IC_50_ (concentration to achieve 50% inhibition of the target) were reduced from 5 µM to 7 nM and from 25 ng/mL to 6 ng/mL, respectively, the potential clinical implications being increased effectiveness of 5-Fluorouracil and Bevacizumab. However, the mechanism for the effect of MNTX involved a membrane-bound phosphatase acting on Src downstream of VEGFR. Together with the fact that naloxone and naltrexone did not replicate the effect of MNTX, these results show that the synergistic effect of MNTX on endothelial cells is non-µopioid receptor mediated (53). A subsequent *in vitro* study from the same group showed similar synergy between MNTX and the mTOR inhibitors Temsirolimus and Rapamycin, reducing both of their IC_50_ for VEGF-induced proliferation and migration of endothelial cells. Furthermore, inhibition of tyrosine phosphate activity blocked this synergy, consolidating that the mechanism of synergy is non-µ opioid receptor mediated (54). Another study indicated MNTX potentiated the effect of the anti-tumour drug docetaxel in gastric cancer cells. In this study, growth inhibition induced by OGF was antagonised by MNTX, releasing the cells from dormancy and making them susceptible to docetaxel. Therefore, MNTX without docetaxel actually enhanced cell growth (55). Low dose naltrexone suppressed proliferation, migration and invasion of HeLa cells by increasing the expression of the tumour suppressor OGFr (56).

Animal studies indicate that the effect of OR antagonists in the whole organism involves complex interactions. Two studies were conducted using μ-opioid receptor agonism or antagonism in murine models inoculated with neuroblastoma tumours. The first study analysed the opioid antagonist naloxone at doses ranging from 5 mg/kg to 20 mg/kg injected subcutaneously either two weeks before or one week after inoculation with the tumour cells. Compared with the control group of mice that received subcutaneous saline injections, both treatment groups experienced a statistically significant increase in survival time, ranging from a 20-61% increase with the higher naloxone doses (15 mg/kg and 20 mg/kg) in both the pre-inoculation and post-inoculation groups. Furthermore, the time to tumour appearance was prolonged by 6-21 days past the median 28 days observed in the control groups. Tumour size was also reduced, however, at the time of death there was no difference in tumour sizes between any of the groups, implying the delay in tumour development as the cause of the size disparity (57). The second study employed the opioid agonist heroin at doses of 3 mg/kg to 15 mg/kg in the same murine model. Importantly, the results of this study showed a statistically significant prolonged mean survival time of 32-39% across all groups when compared to the control, however, no correlation between dose and mean survival time was observed and like in the naloxone study, there was no difference in mean tumour size at the time of death. Interestingly, these anti-tumour effects of heroin were negated by simultaneous administration of naloxone, despite the previous study suggesting its similar effects (58). These studies indicated that both agonism and antagonism of opioid receptor (s) were protective in a rodent experimental tumour model.

Zagon and McLaughlin then exposed the complexity of *in vivo* effects of naltrexone in a study using different dosing regimens: in mice inoculated intradermally with syngeneic neuroblastoma cells, subcutaneous administration of 0.1 mg/kg naltrexone daily led to a reduction in tumour incidence, delay in the time before the tumour appeared and increase in the mean (42%) and median (36%) survival times. However, increasing the dose of naltrexone to 1 mg/kg was less efficacious than 0.1 mg/kg, with the 1 mg/kg group showing no statistically significant reduction in tumour appearance after 29 days, and a survival time comparable to that of the controls. Strikingly, the group receiving 10 mg/kg had statistically significant shorter mean (19%) and median (22%) survival times as well as a reduced time to tumour appearance. Furthermore, this was the only group with a statistically significant difference in the size of tumour at time of death, with an increased mean from 26.2 mm to 32.4 mm (17). How could a low dose of naltrexone be protective, a medium dose have no effect and a high dose be deleterious? The study went on to test the time course of antagonism provided by each regimen and showed that opioid agonism was blocked during 4-6 hours per day in the 0.1 mg/kg group and 24 hours per day in the 10 mg/kg group. Since OR antagonists have been shown to increase MOR expression and endorphin production (59–62), the authors proposed that with the lower naltrexone dosage, μORs were available for activation by endogenous opioid agonists during 19-20 hours per day (17). The authors further reported that the antineoplastic potential of 0.1 mg/kg of naltrexone in a mouse model was not accompanied by a change in metastasis (63). This set of studies underlines the importance of opioid antagonists pharmacokinetics and pharmacodynamics in their anti-cancer effects, and somewhat reconciles the previous findings that both agonists and antagonists offered protection, by suggesting that the antagonists increase the sensitivity to endogenous agonists that are actually protective. This is supported by the antineoplastic activity of β-endorphin shown *in vivo* in models of breast carcinogenesis (64, 65), and antagonised by naloxone (64). Similarly, low dose naltrexone enhanced serum concentrations of beta-endorphin and met-enkephalin and survival rates in dogs with mammary carcinoma (66). Low dose naltrexone was also protective in a murine model of solid Ehrlich carcinoma by increasing the expression of the tumour suppressor OGF receptor (67).

Naltrexone fed to tumour-bearing rats significantly decreased the size of established tumours while tumours continued to grow in control-fed rats when mammary tumours were induced by 7,12-dimethylbenz (a)anthracene (DMBA). After 25 days of receiving a 75 mg/kg naltrexone-supplemented diet, the volume of the mammary tumours was decreased by 23% compared to the control group. Furthermore, tumour regression was observed in 70% of the treatment group. Interestingly, the naltrexone-responsive tumours showed observable amounts of oestrogen and progesterone receptors, contrasting with the unresponsive tumours which were progesterone and oestrogen receptor-negative (68). Moreover, naltrexone administered *per os* to rats in the initiation, promotion or both phases of mammary tumorigenesis upon exposure to DMBA decreased tumour incidence and multiplicity when compared to control (69). Naltrexone’s inhibitory effects on mammary carcinomas were maximised when the diet was supplemented during the promotion phase of carcinogenesis (69).

The implications of the murine studies were inevitably studying of the tumour-suppressive potential of opioid antagonists in a clinical setting. The first of these studies was conducted in 1993 on 21 patients with malignant gliomas being treated with radiotherapy. These patients were randomised to radiotherapy alone (control) and radiotherapy plus Naltrexone. In the combination group a 40% increase in the overall survival at one year was reported (70). Extensive evidence supports that opioid antagonists can effectively be employed in the prevention and treatment of opioid-induced constipation in cancer patients (71). A *post-hoc* analysis was conducted on a cohort of patients with advanced cancer being treated with MNTX for opioid-induced constipation. The difference in the median overall survival was statistically significant favouring those being treated with MNTX. Comparison with seriously ill, non-cancer patients in the same study that had no increase in the median overall survival, hints at potential anti-cancer effects of MNTX (72).

## Evidence that Opioids May Influence Cancer Outcomes *via* Other Receptors

In this last section, we highlight the growing awareness that some of the effects of opioids on cancer may not be mediated by the classical μ, δ or κOR. The role of Toll-like receptor 4 (TLR4) in this context has been reviewed elsewhere (73). In brief, TLR4 is a major player of the innate immune response and is expressed by immune cells of the tumour microenvironment as well as cancer cells (30). TLR4 signalling in cancer cells increases their ability to invade (74, 75). Chronic TLR4 activation promotes an inflammatory environment conducive to carcinogenesis, but TLR4 is necessary to the elimination of dying cancer cells upon radio or chemotherapy (76) and perioperative treatment with a TLR4 agonist in rats and mice reduces cancer metastasis (77). Some opioids, including opioid metabolites or isomers inactive at ORs, have been shown to weakly activate TLR4 (13, 16) and a range of opiates, both agonists and antagonists of ORs, prevent activation of TLR4 by its natural ligand lipopolysaccharide (16, 78). From these interactions, the net effect of opioids acting on TLR4 on the course of cancer cannot be predicted.

The literature offers insight into non OR-mediated effects of opioids that are relevant to cancer. Fentanyl was recently shown to inhibit acute myeloid leukemia cell growth synergistically with cytarabine *via* an opioid receptor-independent mechanism of suppression of Ras and STAT5 pathways (79). A recent clinical study evaluated the interaction between opioid dose administered intraoperatively, canonical oncogenic pathway gene mutation in lung tumours, and recurrence. Alterations of the Wnt and Hippo pathways were associated with improved 5-year recurrence-specific survival with increasing opioid exposure (80). The signalling events linking opioids to these known oncogenic pathways remain to be explored. Opioid-binding protein/cell adhesion molecule-like (OPCML) is a GPI-anchored protein functioning as a tumour-suppressor gene that attenuates multiple receptor tyrosine kinases (81). It was discovered based on an opioid binding strategy of protein purification from the brain and proposed to modulate OR expression and function. Importantly, its expression was reduced in neuronal cells treated by δOR agonists (82). Lastly, we included the OGFr in our search for evidence of ORs expression in cancer even though it is not a classical opioid receptor. The nuclear membrane receptor OGFr, which responds to the endogenous opioid peptides OGF or met-enkephalin, is a known negative regulator of cell proliferation, which controls, amongst other processes, tumour growth and angiogenesis (83). OGFr responds to naltrexone resulting in suppression of cell proliferation, and upregulation of the OGFr (84).

## Concluding Remarks

Our review focused on ORs to highlight the complexity of the role of opioids in cancer. Although the literature points to an overexpression of ORs in cancer, and experimental manipulation of OR expression overall seems to correlate with cancer aggressiveness, the view that opioid agonists promote, and opioid antagonists prevent cancer is oversimplistic. The literature linking μOR mutations that impair receptor function to cancer risk or prognosis in patients does not, to date, offer a clear picture. Studies evaluating the effect of μOR antagonists suggest a protective action in the context of cancer, however the protection they afford is much more nuanced than merely antagonising the ORs; the pharmacokinetics and pharmacodynamics of antagonists and endogenous or exogenous agonists, and feedback on receptor expression, must be evaluated. Lastly, future studies investigating the effect of opioids on non-opioid receptors may contribute to elucidating the role of opioids in cancer.

## Author Contributions

Writing—original draft preparation, CS, ZS, and M-OP. Writing—review and editing, CS, ZS, M-OP, and MH. Supervision, M-OP. Clinical validation, MH. All authors have read and agreed to the published version of the manuscript.

## Conflict of Interest

MH has received research funding from CSL Behring, ZonMw, the Society of Cardiovasular Anesthesiologists (SCA) and the European Association of Cardiothoracic Anaesthesiology (EACTA); and has received compensation from Eurocept Pharmaceuticals BV and IDD for service as a consultant.

The remaining authors declare that the research was conducted in the absence of any commercial or financial relationships that could be construed as a potential conflict of interest.

## Publisher’s Note

All claims expressed in this article are solely those of the authors and do not necessarily represent those of their affiliated organizations, or those of the publisher, the editors and the reviewers. Any product that may be evaluated in this article, or claim that may be made by its manufacturer, is not guaranteed or endorsed by the publisher.
